# The Value of Emotion: How Does Episodic Prospection Modulate Delay Discounting?

**DOI:** 10.1371/journal.pone.0081717

**Published:** 2013-11-28

**Authors:** Lei Liu, Tingyong Feng, Jing Chen, Hong Li

**Affiliations:** 1 School of Psychology, Southwest University, Chongqing, China; 2 Key Laboratory of Cognition and Personality, Ministry of Education, Chongqing, China; 3 School of Psychology, Beijing Normal University, Beijing, China; 4 School of Psychology, Liaoning Normal University, Dalian, China; University of Pennsylvania, United States of America

## Abstract

**Background:**

Humans often show impatience when making intertemporal choice for monetary rewards, preferring small rewards delivered immediately to larger rewards delivered after a delay, which reflects a fundamental psychological principle: delay discounting. However, we propose that episodic prospection humans can vividly envisage exerts a strong and broad influence on individuals' delay discounting. Specifically, episodic prospection may affect individuals' intertemporal choice by the negative or positive emotion of prospection.

**Methodology/Principal Findings:**

The present study explored how episodic prospection modulated delay discounting by emotion. Study 1 showed that participants were more inclined to choose the delayed but larger rewards when they imaged positive future events than when they did not image events; Study 2 showed that participants were more inclined to choose the immediate but smaller rewards when they imaged negative future events than when they did not image events; In contrast, study 3 showed that choice preferences of participants when they imaged neutral future events were the same as when they did not image events.

**Conclusions/Significance:**

By manipulating the emotion valence of episodic prospection, our findings suggested that positive emotion made individuals tend to choose delayed rewards, while negative emotion made individuals tend to choose immediate rewards. Only imaging events with neutral emotion did not affect individuals' choice preference. Thus, the valence of imaged future events' emotion might play an important role in individuals' intertemporal choice. It is possible that the valence of emotion may affect the changed direction (promote or inhibit) of individuals' delay discounting, while the ability to image future events affects the changed degree of individuals' delay discounting.

## Introduction

Humans often show impatience when making intertemporal choice for monetary rewards, preferring small rewards delivered immediately to larger rewards delivered after a delay. This tendency reflects a fundamental psychological principle: delay discounting (temporal discounting) [1], [2]. For example, individuals might prefer $100 available now to $120 available in 2 months, because $120 available, delivered after 2 months delay, is less attractive than $100 available now. Numerous researches indicated that the devaluation of a future reward was as a function of the time to the delivery of that reward [3]–[5]. Over time, this discounting pattern has been repeatedly described to fit a hyperbolic function [1], [6], and then, it has been described as a quasihyperbolic function [7]. The degree of delay discounting varies considerably between individuals [8] and correlates with individual differences in real-world behavior. However, humans can engage in episodic prospection, and it has been suggested that the episodic prospection can reduce Reward delay discounting [9], [10].

Humans can vividly envisage possible future episodes (also referred to as episodic prospection or prospective thinking) [11]–[13]. There are converging evidences which indicated episodic prospection exerted a strong and broad influence on individuals' delay discounting [9], [14]–[16]. In previous studies, Loewenste (1987)[14] thought that episodic prospection is a source of utility, which can increase or lessen the subjective value of future rewards. Recently, Peters and Büchel (2010)[9] found that episodic prospection reduced the rate of delay discounting through a modulation of neural decision-making and episodic prospection networks. In addition, Benoit et al. (2011) [16] showed that the human faculty of episodic prospection effectively motivated decisions in the present which will only be advantageous in the future. For example, when you order a piece of cake at the beginning of a dinner, you are evaluating the pleasure you will receive on the basis of your current hunger. Because there is an asymmetry between the value of the cake when you make the choice and the value when you eat it, eating the cake may be far less valuable than expected before, which can lead individuals to different decisions [17]. Thus, this mechanism on how episodic prospection affects intertemporal choice is important to understand.

Numerous researches showed that emotion guided human decision making [18]–[20]. Previous researches have shown that even incidental emotion which is unrelated to the decision at hand can have a significant impact on judgment and choice [18], [21]. Loewenstein and Lerner (2003) [19] have pointed out that many decisions involve predictions of future feelings. In a word, the emotion of episodic prospection may play an important role in decision making. Clore and Huntsinger (2007)[20] suggested that the emotion-as-information hypothesis explained how decision making was affected by assuming that emotion served as compelling information about value. Positive affect signals that the object of judgment is valuable, leading to a positive evaluation, and negative affect signals that it lacks value, leading to a negative evaluation; and then positive or negative value might influence individual's different decision making. This is consistent with the somatic marker hypothesis. The somatic marker hypothesis by proposed by Bechara and Damasio [22] showed that when pondering a decision, separate thoughts trigger a positive or negative somatic state. Positive and negative somatic states induce distinct physiological patterns which exert different effects on decisions. That is, positive somatic state leads a positive evaluation, whereas negative somatic state leads a negative evaluation. Thus, we propose that episodic prospection might modulate the delay discounting by emotion.

Here, we suggest that the emotion-of-episodic-prospection hypothesis is to account for those puzzling features of episodic prospection – how episodic prospection affects delay discounting. Imagined future episodes can trigger specific time-travel experience without the need for deliberate retrieval or construction. Once triggered, they generally activate emotional circuitry, and emotion provides compelling information about the personal value of whatever is in mind at the time. These emotional rewards themselves are outside cognitive control and are appropriate given the situation imagined. In recent study, Benoit et al. (2011) [16] thought that episodic prospection allows for the immediate experience of the envisaged, delayed reward value. Consistent with the account, the emotion-of-episodic-prospection hypothesis suggested episodic prospection can trigger specific time-travel experience. Inconsistent with the account, although the immediate time-travel experience was triggered, they generally activate emotional circuitry, and prospective emotion (negative or positive) may play the role of a reducing or a boosting on patience in intertemporal choice. In this view, imagination may play the role of a reducing or a boosting on patience by associating our plans with negative or positive non-controlled emotion [10]. Imaging good future thing could make us far-sighted (e.g., Imaging vacation Paris will come) [9], whereas imaging dire future thing could make us short-sighted (e.g., Imaging an electric shock will come) [16]. Thus, we suppose that episodic prospection may modulate delay discounting by emotion. The positive emotion of prospection could inhibit individuals' delay discounting, yet the negative emotion of prospection could promote individuals' delay discounting. However, the ability to image future events could affect the changed degree of individuals' delay discounting.

In the work we reported here, we studied how episodic prospection modulated delay discounting by emotion. In Experiment 1, we first asked participants to complete an intertemporal choice without the image task, and then they completed the homogeneous intertemporal task when they imaged positive future events; Experiments 2 and 3 were identical to Experiment 1 except for the followings: in Experiment 2, instead of imaging positive future events in Experiment 1, we asked participants to image negative future events; in Experiment 3, they imaged neutral future events. Based on the emotion-of-episodic-prospection hypothesis, we expected that participants would be more inclined to choose the delayed but greater rewards when the intertemporal choice task involved the positive episodic cue; participants would be more inclined to choose the immediate but smaller rewards when the intertemporal choice task involved the negative episodic cue. However, the preference of participants' choice would not change when the intertemporal choice task involved the neutral future events.

## Experiment 1

Experiment 1 examined whether positive episodic prospection modulated individuals' delay discounting. By comparing experimental condition (imaging positive future events) with control condition (no imaging events), we tested whether positive episodic prospection decreased individuals' preference for immediate rewards.

### Participants

Participants were 32 undergraduate students who were from a Chinese university (M = 20.62 years, SD = 1.92 years, age range  = 18–25 years; 17 females). Participants were compensated with payments based on their selections. Participants provided their verbal informed consent to participate in this study. Present study did not cause participants adversely physiological and psychological reaction, and it only tested simple behavior responses. So the institutional review board waived the need for written informed consent from the participants. The ethical approval was obtained for the experiments reported in this study: Approved by the Institutional Review Board of the Southwest University - Approval number SWU20120311.

### Materials and Procedures

Before the behavioral test, each participant was thoroughly familiarized with the tasks and 10 different positive future events. A positive future event was defined as something participants are looking forward to (e.g., winning an award, birthday party) [25]. Because all the subjects were college students, prospective events that college students are familiar with were selected. These positive events were very relevant with the college students and happen frequently. Participants carefully imagined each event firstly, and then they rated these events on seven-point scales for valence and personal relevance. For the behavioral test, five events were chosen that were positive and high in personal relevance for each participant (valence: *M* = 6.73, *SD* = 0.445; personal relevance: *M* = 6.18, *SD* = 0.470). Given that subjects were familiar with these events and they had imaged the events, it was easy for subjects to image these events in intertemporal choice task. After the experiment, subjects were asked whether they really tried to image these events, and all of them said they did. Furthermore, we also asked the participants how they image, and what their feelings are when they were doing the experiments. The results of the subjective report showed that the details of their imagination are consistent with the positive emotion of our experiment manipulation.

The task was a delay discounting procedure based on a previously used task [26]. A white fixation was shown for 800 ms, signaling the start of the trial. After a 300–500 ms random blank, each participant first evaluated a series of binary choices between ¥d today or ¥d′ in one week, half one month, or one month (e.g.,¥6 today or¥9 in one week). For each trial, the immediate RMB amount (¥d) was drawn randomly from a Gaussian distribution with mean ¥20 and standard deviation¥10, clipped to give a minimum of ¥10 and a maximum of ¥30. The percent difference in renminbi yuan (RMB) amounts between the two rewards ((¥d′−¥d)/¥d) was selected from the set {10%, 15%, 25%, 35%, 50%, 75%, 95%, 125%}. There was 4000 ms to evaluate their choice preferences. Following another 600–800 ms random blank, participants made their decision, in which participants pressed the square to choose the immediate reward and the triangle to choose the delayed reward. After participants confirmed their choices, another 800 ms fixation signaled the start of the next trial. That the square and triangle represented the immediate reward or the delayed reward was counterbalanced between participants.

**Figure 1:**
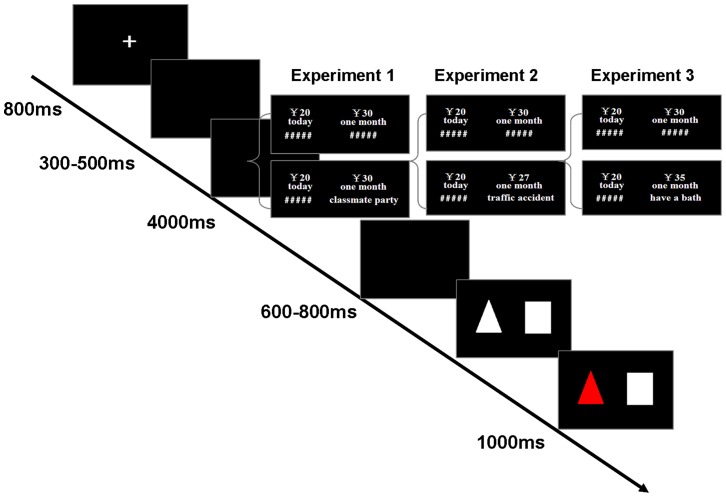
Behavioral Task: subjects made repeated choices between a smaller but immediate reward and a larger but delayed reward. In the control condition, only amount and waiting time were shown, whereas in the experimental condition, a verbal episodic tag (Experiment 1: positive events; Experiment 2: negative events; Experiment 3: neutral events with neutral emotion) was also shown, besides amount and waiting time.

In short, participants first completed the intertemporal task for 96 trials in control condition, in which they did not image future events. Then, they completed the homogeneous intertemporal task, in which they were asked to image positive future events. Each trial consisted of the choice between a smaller but immediate reward and a larger but delayed reward. In experimental condition, a verbal episodic tag (see Figure 1) was also shown, besides amount and waiting time. The verbal episodic tag indicated that subjects had to image the future events which would happen at the respective day of delayed reward delivery. In control condition, only amount and waiting time were shown. No events had been presented, and “#####” tags would be shown. Thus, participants were not instructed to use imagery to envision future events. Furthermore, participants were told that image events were independent of the choice task.

Participants completed a practice session that only involved trials from the control condition. In addition, participants were instructed that they would receive one of their choices (randomly selected from the set of all of their choices) as their payments at the end of the experiment. Next, they were explicitly told that based on this payment scheme. Thus, they should make each choice as though it was the one they were actually going to receive. At the end of the experiment, participants implemented a computer lottery. The procedure could randomly extract a numeral between 1 and 192 in which the numeral indicated the specified trial that determined how much payoff participants got and when participants got their payoff. For example, if participant chose immediate reward in the trial, the money was available at the end of the experiment; if participant chose delayed reward in the trial, the money was available one week later (half one month, or one month).Percentage of immediate rewards (%) chosen in each condition was as the ratio of interest [27]–[28]. The higher the ratio of interest, the greater the value participants assigned to preferring immediate rewards.

### Results and Discussion

**Figure 2:**
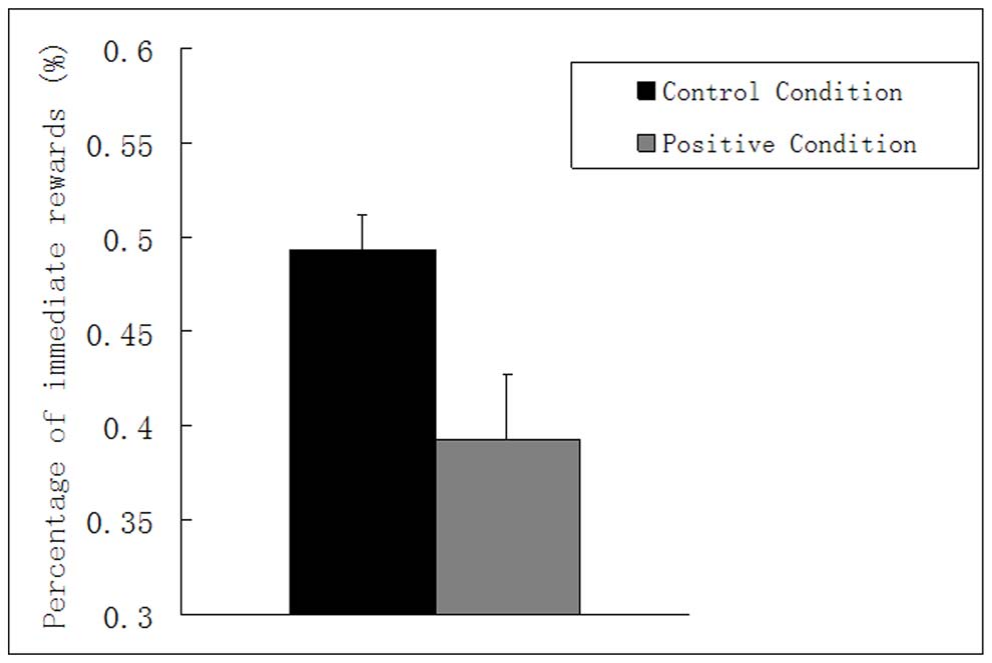
Results from Experiment 1: mean percentage of immediate reward as a function of the control condition (no imaging) and the experimental condition (imaging positive events). Error bars indicate standard errors of the mean.

We tested a Paired-samples T test with condition (experimental condition, control condition) as the paired factor. As expected, participants when they needed to image positive future events were more inclined to choose delayed rewards (M = 39.28%, SE = 3.42%, experimental condition) than when they did not image events (M = 49.30%, SE = 1.87%, control condition), t(31) = 3.114, p = .004, *d*  = 0.67 (see Figure 2). In other words, participants when imaging positive future events were more likely to wait a larger payment in a delayed time rather than to accept a smaller payment now. Positive future thinking seemed to make people patient in a manner, which could make them foresighted.

## Experiment 2

In Experiment 2, we further test the emotion-of-episodic-prospection hypothesis by examining whether negative episodic prospection promotes individuals' delay discounting. We did this by taking similar paradigm we used in Experiment 1 to compare experimental condition (imaging negative events) with control condition (no imaging events).

### Participants

A total of 31 undergraduate students from a Chinese university (M = 20.74 years, SD = 1.98 years, age range  = 18–25 years; 15 females) were included in the test and the payments of participants were identical to those given in Experiment 1. Participants provided their verbal informed consent to participate in this study. The study was approved by the Institutional Review Board of the Southwest University (same to experiment 1).

### Materials and Procedures

Similarly, participants were thoroughly familiarized with the tasks and 10 different negative future events before the behavioral test. A negative event was defined as an experience they would prefer to avoid (e.g., getting ill, failed in the exam) [25]. Prospective events that college students are familiar with were selected. These negative events were very relevant with the college students and happen frequently. Participants carefully imagined each event and rated these events on seven-point scales for valence and personal relevance. For the behavioral test, five events were chosen that are negative and high in personal relevance (valence: *M* = 1.35, *SD* = 0.480; personal relevance: *M* = 5.65, *SD* = 0.589). Similar to the experimental 1, the results of the subjective report showed that the details of their imagination are consistent with the negative emotion of our experiment manipulation.

The task of Experiment 2 was identical to those used in Experiment 1. Participants first completed the intertemporal task in control condition, in which they did not image future events. Then, they completed the homogeneous intertemporal task, in which they were asked to image negative future events (see Figure 1).

### Results and Discussion

**Figure 3:**
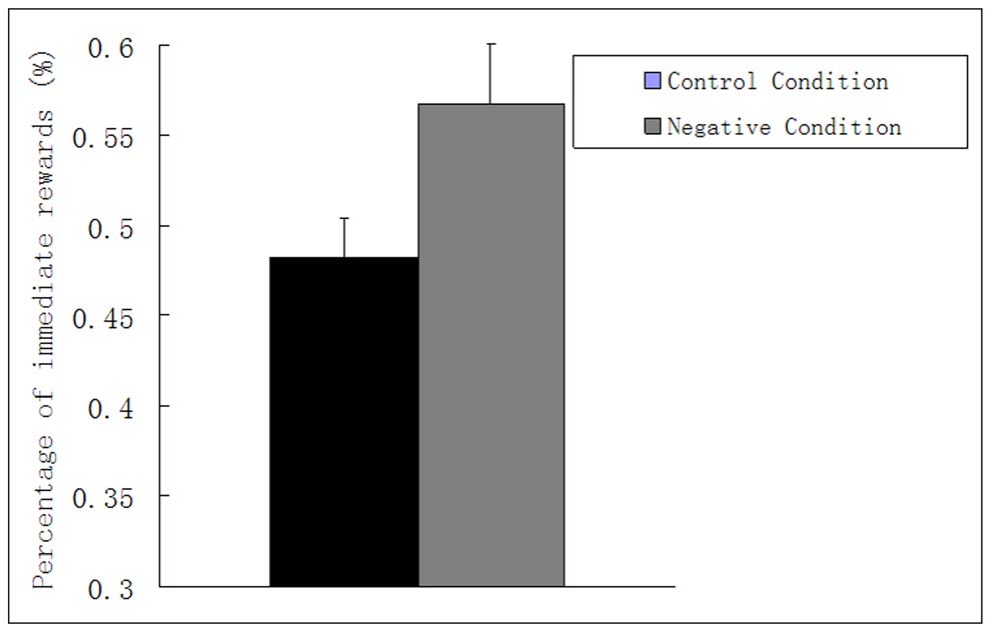
Results from Experiment 2: mean percentage of immediate reward as a function of the control condition (no imaging) and the experimental condition (imaging negative events). Error bars indicate standard errors of the mean.

Similar to the statistics method in Experiment 1, we tested a Paired-samples T test with condition (experimental condition, control condition) as the paired factor. The results showed that participants were more inclined to choose immediate rewards (M = 56.78%, SE = 3.29%, experimental condition) when they needed to image negative future events than when they did not image future events (M = 48.27%, SE = 2.15%, control condition), t (30) = −3.544, p = .001, *d*  = 0.56 (see Figure 3). That is, participants when imaging negative future events were more likely to accept a smaller payment now rather than to wait a larger payment in a delayed time. Negative future thinking seemed to make people impatient in a manner, which could put their economic interest at ‘myopic’ decision making.

## Experiment 3

By now, we have shown that imaging positive and negative future events affected individuals' delay discounting. So can imaging neutral future event also influence individuals' choice preference? In Experiment 3, we test whether imaging future events with neutral emotion can influence individuals' delay discounting.

### Participants

A total of 30 undergraduate students from a Chinese university (M = 21.48 years, SD = 1.95 years, age range  = 18–25 years; 16 females) were recruited to participate in the study. They were given payments for their participation. Participants provided their verbal informed consent to participate in this study. The study was approved by the Institutional Review Board of the Southwest University (same to experiment 1).

### Materials and Procedures

Before the behavioral test, participants were thoroughly familiarized with the tasks and 10 different neutral events. A neutral event was defined as repetitive tasks that participants perform everyday (or at least several times a week) and that are emotionally neutral (e.g., having a bath, washing the clothes) [25]. Similarly, prospective events that college students are familiar with were selected. These neutral events were very relevant with the college students and happen frequently. Participants carefully imagined each event and rated these events on seven-point scales for valence and personal relevance. For the behavioral test, five events were chosen that are neutral and high in personal relevance (valence: *M* = 3.98, *SD* = 0.614; personal relevance: *M* = 6.27, *SD* = 0.775). Similar to the experimental 1, the results of the subjective report showed that the details of their imagination are consistent with the neutral emotion of our experiment manipulation.

The task of Experiment 3 was identical to those used in Experiment 1 and Experiment 2. Participants first completed the intertemporal task in control condition, in which they did not image future events. Then, they completed the homogeneous intertemporal task, in which they were asked to image neutral future events (see Figure 1).

### Results and Discussion

**Figure 4:**
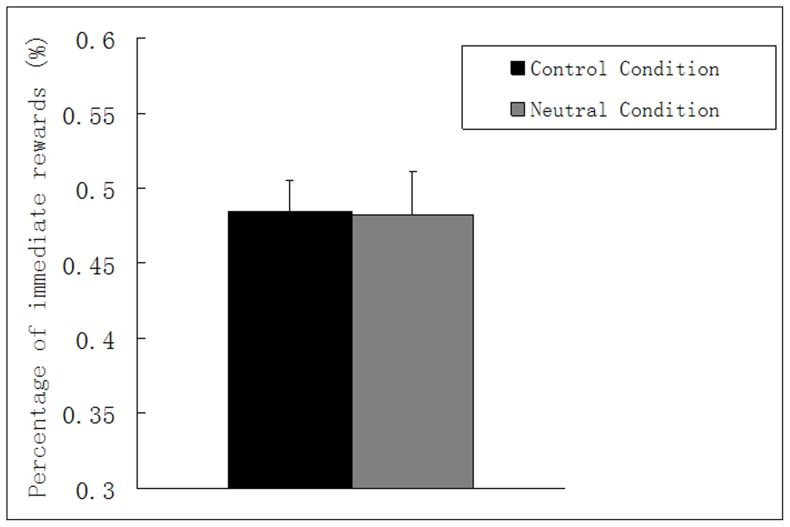
Results from Experiment 3: mean percentage of immediate reward as a function of the control condition (no imaging) and the experimental condition (imaging neutral events). Error bars indicate standard errors of the mean.

Similarly, we tested a Paired-samples T test with condition (experimental condition, control condition) as the paired factor. The results showed that there was no significant difference about choice preference between experimental condition (participants needed to image neutral events, M = 48.27%, SE = 2.85%) and control condition (participants did not image future events, M = 48.45%, SE = 2.12%), t (29) = 0.100, p = .921, *d*<*0.01* (see Figure 4). Thus, neutral episodic prospection with neutral emotion did not affect the participants' choice preferences.

In addition, two-way ANOVA on the valence of prospective emotion (Positive (Experiment 1) vs. Negative (Experiment 2) vs. Neutral (Experiment 3)) and the type of conditions (Control condition vs. imaging condition) revealed that there was a significant interaction effect between the two factors (F (2, 90) = 6.040, p = .003, *η^2^_partial_* = 0.222). After performing simple effect analysis, the following results were obtained: In the control conditions, there were no significant differences among three experiments (p>.050). This can be regard as the homogeneity of participants in the three experiments. In the imaging conditions, participants in Experiment 1 (positive condition) preferred more delayed rewards than those in the Experiment 2 (negative condition) (p<.001) and those in the Experiment 3 (neutral condition) (p = .049). In contrast, participants in the Experiment 2 (negative condition) preferred more immediate rewards than those in the Experiment 1 (positive condition) (p<.001) and those in the Experiment 3 (neutral condition) (p = .046). Together these findings, our results showed that the positive emotion prospection made participants to prefer more delayed rewards than neutral emotion prospection, whereas negative emotion prospection made participants to prefer more immediate rewards than neutral emotion prospection.

## General Discussion

The present research provides novel insights into how episodic prospection modulated delay discounting. Our findings showed episodic prospection might modulate delay discounting by emotion. Study 1 showed that participants were more inclined to choose the delayed but larger rewards when they imaged positive future events than when they did not image events; Study 2 showed that participants were more inclined to choose the immediate but smaller rewards when they imaged negative future events than when they did not image events; In contrast, study 3 showed that the choice preferences of participants when they imaged neutral future events was the same as when they did not image events. These results suggested that episodic prospection could modulate delay discounting by emotion (Experiment 1 and Experiment 2), and only future thinking (with neutral emotion) could not change delay discounting (Experiment 3). Positive emotion prospection could inhibit individuals' delayed discounting, whereas negative emotion prospection could promote individuals' delay discounting.

We offer an explanation for how episodic prospection affects temporal discounting. Episodic prospection may provide a motivational ‘brake’ that counters natural dispositions towards short-termist, ‘myopic’ decision making or long-termist, ‘self-control’ decision making [10]. Furthermore, a way that prospection abilities affect decision-making is through emotion, in which emotions can be generated by imagining cognitive processes associated to prospects. Recent neuroscientific research has focused on how anticipated emotions change the utility of prospects [23], [24]. For example, Berns et al. (2006)[24] reported that subjects preferred to receive an electric shock immediately rather than after a given amount of time; in some cases, subjects preferred a stronger electric shock immediately rather than waiting for a weaker one. According to the experimenters, the subjects assigned negative utility to waiting, because they anticipated their negative emotional state during the waiting time [17]. Thus, episodic prospection may affect individuals' decision making by the negative or positive emotion.

The findings supported the emotion-of-episodic-prospection hypothesis. According to the hypothesis, Imagined future episodes can trigger specific time-travel experience without the need for deliberate retrieval or construction. Once triggered, they generally activate emotional circuitry, and emotion provides compelling information about the personal value of whatever is in mind at the time. These emotional rewards themselves are outside cognitive control and are appropriate given the situation imagined. Thus, episodic prospection may be functional to the extent that it provides emotions which supply compelling information about the personal value of whatever is in mind [10]. Moreover, some prospective emotions are adaptive-motivation devices and connected to episodes constitute self-persuasion devices [10], [29]. That is, Imagination may play the role of a reducing or a boosting on patience by associating our plans with negative or positive non-controlled emotion.

Why dose that positive emotion inhibit and negative emotion promote individuals' delay discounting? According to the affect-as-information hypothesis, emotion provides compelling information about the personal value of whatever is in mind at the time. In the case of judgment, value might be assigned to the object of judgment. In such cases, positive affect signals that the object of judgment is valuable, leading to a positive evaluation, and negative affect signals that it lacks value, leading to a negative evaluation[20]. In the case of processing, by contrast, value might be assigned to the person's own cognitions and inclinations. Thus, positive emotion or negative emotion influences the content of thought in which emotion serves as information about the value of whatever comes to mind. Thus, our findings demonstrated that emotion might play an important role in intertemporal choice. Participants when they imaged positive future events would be more inclined to choose the delayed but larger rewards. However, participants when they imaged negative future events would be more inclined to choose the immediate but smaller rewards.

The present findings may also provide an insight into the theoretical significance of isolating causes of impatience which can lead to a wide range of suboptimal decisions, from under-saving for retirement to problems with self-control such as overeating and addiction. Our results showed that the negative emotion of episodic prospection promoted impatience behavior; Furthermore, the positive emotion of episodic prospection highly inhibited impatience behavior. So far, a few recent studies have examined specific training procedures aimed at reducing impulsive discounting [15], [30]. Our findings may help to provide a way that can reduce impulsive discounting or increased future-minded choice, such as, imaging positive future events.

This study had some limitations. First, between the three of experiments, we used a between-subjects design rather than within-subjects in order to rule out order effect. Although individual differences may affect our results, our results showed that there were no significant differences among control conditions, reflecting the homogeneity of participants in the three control conditions. Second, although the presentation order of control condition and experiment condition was not counterbalanced in each experiment, there were the same results by comparing the three experiments longitudinally. The results showed that positive emotion made participants to prefer more delayed rewards than neutral emotion prospection, whereas negative emotion made participants to prefer more immediate rewards than neutral emotion prospection. Thus, the consensus results indicated the effectiveness of the manipulation of the experiments.

Third, in our experiments, participants were randomized. Before they did the experiment, their emotion should be neural. Even if their emotions have some changes when they were doing the experiment, these changes is also due to imagine future events. However, imaging future events that are associated with emotion might induce the emotional change in current. The present study did not support that the behavioral changes in delayed discounting were due to prospecting future emotional events rather than the present emotional change, which need future research to explore. Finally, this study is the first to explore how episodic prospection modulated delay discounting by emotion. However, because we did not measure directly the change of emotion, the present study can not provide evidence to support our explanation. Previous research showed that humans seem able to anticipate pleasure or displeasure associated with a future out come just by imagining it [16]–[17], [23], [31]. Thus, we think participants' emotions could be changed after imaging my study materials.

By manipulating the emotion valence of episodic prospection, our findings suggested that positive emotion made individuals tend to choose delayed rewards, while negative emotion made individuals tend to choose immediate rewards. Only imaging events with neutral emotion did not affect individuals' choice preference. This is in line with many emotion studies which indicated that positive emotion led to a positive evaluation, and negative emotion led to a negative evaluation [32]–[33]. Previous studies showed that the ability of imaging future events reduced individuals' delay discounting [9], [16] in which the most of future events were positive events, but our findings suggested that the valence of imaged future events' emotion played an important role in individuals' intertemporal choice. It is possible that the valence of emotion may affect the changed direction (promote or inhibit) of individuals' delay discounting, while the ability to image future events affects the changed degree of individuals' delay discounting.
